# Prostate specific antigen test uptake: a cross sectional study on elderly men in Western Iran

**DOI:** 10.1186/s12877-020-01710-9

**Published:** 2020-08-24

**Authors:** Mehdi Mirzaei-Alavijeh, Farzad Jalilian, Laleh Solaimanizadeh, Abdollah Saadatfar, Shima Khashij, Razieh Pirouzeh, Farzaneh Solaimanizadeh

**Affiliations:** 1 Social Development & Health Promotion Research Center, Health Institute, Kermanshah University of Medical Sciences, Kermanshah, Iran; 2 Lifestyle Modification Research Center, Imam Reza Hospital, Kermanshah University of Medical Sciences, Kermanshah, Iran; 3 Department of Nursing, Faculty of Nursing and Midwifery, Bam University of Medical Sciences, Bam, Iran; 4 Department of Urology, School of Medicine, Kermanshah University of Medical Sciences, Kermanshah, Iran; 5 Ministry of Health and Medical Education, Tehran, Iran

**Keywords:** Prostate specific antigen test, Elderly, Benefits, Cues to action, Iran

## Abstract

**Background:**

Prostate cancer (PCa) is the second most common malignancy in men worldwide and the incidence rate of PCa has been increasing in recent years. The aim of the current study was to determine beliefs elderly men towards prostate-specific antigen (PSA) test uptake.

**Methods:**

This cross-sectional study conducted among 352 elderly men (60–74 years old age) in the west of Iran. The Health Belief Model (HBM) was applied as a study framework to evaluation of beliefs towards PSA test uptake. Data were analyzed by SPSS version 16 using appropriate statistical tests including t-test, chi-square, bivariate correlations, and logistic regression at 95% significant level.

**Result:**

The mean age of participants was 65.55 years [SD: 3.90]. Almost 16.9% of the elderly men had uptake PSA during last year. There was significant association between PSA test uptake with older age (*P* = 0.013), better economic status (*P* = 0.023), higher education level (*P* = 0.004), positive family history of prostate cancer (*P* = 0.018), and number of family members more than four (*P* = 0.032). The best determinants predictors for PSA test uptake were cues to action [OR: 1.967 and 95% CI: 1.546, 2.504], perceived severity [OR: 1.140 and 95% CI: 1.008, 1.290], and perceived benefits towards PSA test uptake [OR: 1.133 and 95% CI: 1.024, 1.253].

**Conclusions:**

It seems that development of health promotion programs to increase cues to action and positive beliefs toward PSA test uptake and also perceived treat about side effect of PCa could be beneficial to increase PSA test uptake.

## Background

Prostate cancer (PCa) is the second most common malignancy in men worldwide, counting 1,276,106 new cases and causing 358,989 deaths (3.8% of all deaths caused by cancer in men) in 2018 [1]. The incidence rate of this cancer has been increasing in recent years [2]. The growing rate of PCa cases has also been evident in Iran for the last 10 years [3]. Despite major differences in the incidence rate of this malady, PCa could mainly be considered as an illness related to men who are older than 65 years since more than 75% of its new cases are diagnosed in men older than 65 years [4]. However, other causes include racial differences, genetic and environmental factors, family history, hormonal changes related to aging, poor nutrition (especially consuming monounsaturated fats), smoking or alcohol consumption [5]. Availability and access to diagnostic and health-care services as well as recommendations regarding PCa testing may be usefulness of the results in order to reduce incidence and mortality rates [2]. American cancer society recommended that men over 50 years of age should receive a PCa screening test; serologic test for assessing prostate-specific antigen (PSA) level is the most important method, which is also the most practical one, easiest and most sensitive detection test (97% specificity and 67% sensitivity) [6]. Iran, the private and public sectors both provide health care and treatment services; however, public sector and specially the ministry of health play a more significant role in this regard [7]. About 90% of Iranians covered by some form of health insurance [8], for example, many cancer screening tests at public health centers in Iran are performed for free or are covered by health insurance in the target group population [9]. However, the rate of cancer screening tests in Iranians is low [3, 9]. For encouraging population to uptake screening tests, some studies have emphasized the utilization of fear appeal strategies and interventions which are based on increased knowledge in the framework of prostate cancer prevention programs for health educators [10]. This issue indicates the importance of considering the psychological aspects of participation in cancer screening programs, and, theoretical knowledge of health education experts and utilization of theory-based approaches regarding why people perform or not perform a behavior could guide the experts for designing an effective and efficient educational program [11]. It seems that using cognitive determinants like health belief model (HBM) constructs for develop educational programs can enhance men’s knowledge of PCa, change their health beliefs and improve their behaviors regarding screening programs like PSA. For example, Bilgili et al. [12] conducted a study on 650 Turkish men aged 40 years old and older and showed that strong positive correlation between knowledge and seriousness perception of PCa. Moreover, several studies have been carried out to assess the HBM determinants predict the PCa screening behaviors and indicated that the when men perceive the benefits of screening behavior, they can defeat the barriers and costs of the behavior through believing in their ability to perform these behaviors (perceived self-efficacy) and uptake test [13, 14]. Furthermore, perceived susceptibility and perceived severity of people refers to their belief based on their vulnerability to PCa [15, 16]. The HBM is one of the most commonly used models in the field of PCa screening behaviors [12–17]. The objective of current study was to determine prevalence and determinates related to uptake PSA test among sample of Iranian elderly men based on the HBM.

## Methods

### Study design and study population

This cross-sectional study was conducted on 352 elderly men (60–74 years old age) in the Kermanshah Province in the western part of Iran. Kermanshah is the capital of Kermanshah Province, is located in western Iran and close to Iraq; according to the last census, its population is 946,681 (2019 estimate 1,046,000); a majority of the population language is Kurdish. Kermanshah has a moderate and mountainous climate [18]. To register the participants and collect the data, the following steps were done. At first, different parts of the city were divided into eight regions based on the municipalities and one health center was selected from each region. Subsequently, elderly men referred to the health centers for taking health care, were randomly selected into the current study voluntarily. Men aged 60 to 74 years, and speak Kurdish fluently were eligible to participate in this study. The sample size was calculated at 95% significant level according to the results of a pilot study. According to the PSA test uptake rate among elderly men in the pilot study (which is 26% and taking into account the 5% error), the required sample size was estimated at 352 people. Among 352 elderly men invited to participate in our study, 320 elderly men signed the consent form and voluntarily agreed to participate in the study, which has been approved by the research ethics committee at the Kermanshah University of Medical Sciences, Kermanshah, Iran (IR.KUMS.REC.1398.431). The response rate was 90.9%.

### The study tool

The questionnaire consists of three parts: 7 questions for demographic factors, 1 item about PSA test uptake and 23 items for HBM determinants. The designed questionnaire has been uploaded as a supplementary file.

### Demographics

The demographics variables assessed in current study included: age (year), marital status (married, single), economic status (weak, middle, good), education (primary school, secondary school, high school, and academic), family member size (1–4 number, More than 4 number), health insurance (yes, no), and history of a family person who has had PCa (yes, no).

### HBM theoretical determinants

The items which assessed determinants of the HBM were derived from the questionnaires of beliefs towards PCa screening behaviors [12–17] and in accordance with expert panel comments. The expert panel included five health educators, two health policymakers, two health services manager, one public health expert, and two urologists. There were 23 items which measured the six determinants of 1) perceived benefits, 2) perceived barriers, 3) perceived susceptibility, 4) perceived severity, 5) perceived self-efficacy, and, 6) cues to action. In order to facilitate participants’ responses to the items, all items were standardized to a five-point Likert scale, ranging from 1 (strongly disagree) to 5 (strongly agree) was used to measure the perceived benefits, perceived barriers, perceived susceptibility, perceived severity, and perceived self-efficacy. Furthermore, for measured the cues to action was used yes or no. The face validity of the questionnaire was evaluated qualitatively. Thus, face-to-face individual interviews were held up with 12 experts, their comments analyzed and the necessary modification performed. In addition, prior to conducting the main project, a pilot study was conducted to assess the internal consistency of the questionnaire and estimating the sample size. The pilot study subjects were 30 elderly men, similar to those who participated in the main study. Cronbach’s Coefficient Alpha was used to estimate the internal consistency of the various measures. Table 1 shows the HBM scale items.

**Table 1:** The HBM questionnaire items

No	Construct	Item	Cronbach’s Alpha
	**Perceived benefits towards the PSA test uptake**		0.87
1		PSA test uptake will help to diagnose PCa early.	
2		PSA test uptake will help me not worry as much about PCa.	
3		PSA test uptake will decrease my chances of dying from PCa.	
4		PSA test uptake will help me to have a plan for the future about PCa.	
	**Perceived barriers related to PSA test uptake**		0.80
1		PSA test uptake is time-consuming.	
2		I’m afraid of diagnose PCa.	
3		Health center is far from my house to receive PSA test uptake.	
4		PSA test uptake is too embarrassing.	
	**Perceived susceptibility**		0.70
1		It is likely that I will get PCa in the future.	
2		My chances of getting PCa in the next few years are high.	
3		I feel I will get PCa sometime during my life.	
	**Perceived severity**		0.71
1		PCa could seriously affect in my social life.	
2		PCa imposes huge economic costs on my family.	
3		PCa can kill me.	
4		PCa is a serious disease.	
5		Death from PCa is rare.	
	**Perceived self-efficacy**		0.75
	*How confident are you that you can …*		
1		Make an appointment to have a PSA test uptake?	
2		Find the time to have a PSA test uptake?	
3		Get a PSA test uptake even if you are worried about the results?	
	**Cues to action**		0.65
1		Doctors advised me to uptake PSA.	
2		Health care workers encourage me to PSA test uptake.	
3		My family encourages me to PSA test uptake.	
4		How much the PCa death in others affects you to PSA test uptake?	

### PSA test uptake questionnaire

To assess whether or not the subjects had experimented with PSA test uptake, we used one items “Have you PSA test uptake at during last year” which the response category was yes or no.

### Statistical methods

Quantitative variables were expressed as means with SDs, and qualitative/categorical ones as frequencies and percentages. Multivariable logistic regression models were performed to predict study outcomes of PSA. A stepwise backward approach was used to select the independent variables for the final models. Results of logistic models were expressed as ORs with 95% CIs. Bivariate correlations were computed to ascertain the magnitude and direction of the associations between the HBM determinants scores. Independent sample t-test and chi-square were used to assess the relationship between demographics variables and PSA test uptake. The level of significance was (*P* <  0.05). Data were analyzed by the SPSS software for Windows (ver. 16).

## Results

The mean age of respondents was 65.55 ± 3.90 years [95% CI: 65.13, 65.98], ranged from 60 to 74 years. Almost 16.9% of the elderly men had PSA test uptake during last year. There was significant association between PSA test uptake with older age (*P* = 0.013), better economic status (*P* = 0.023), higher education level (*P* = 0.004), positive family history of PCa (*P* = 0.018), and number of family members more than four (*P* = 0.032). More details regarding demographic characteristics of the participants are shown in Table 2.

**Table 2:** Demographic variable and PSA test uptake

		Total	PSA test uptake		***P***-value
		N (%) Mean (SD)	No Mean (SD) N (%)	Yes Mean (SD) N (%)	
Age		65.55 (3.90)	65.31 (3.87)	66.75 (3.84)	0.013
Marital status	Single	32 (10%)	29 (10.9%)	3 (5.6%)	0.321
	Married	288 (90%)	237 (89.1%)	51 (94.4%)	
Economic status	Weak	68 (21.3%)	64 (24.1%)	4 (7.4%)	0.023
	Middle	196 (61.2%)	158 (59.4%)	38 (70.4%)	
	Good	56 (17.5%)	44 (16.5%)	12 (22.2%)	
Educational level	Primary school (grades 0–6)	121 (37.8%)	100 (37.6%)	21 (38.9%)	0.004
	Secondary school (grades 7–9)	109 (34.1%)	99 (37.2%)	10 (18.5%)	
	High school (grades 10–12)	73 (22.8%)	57 (21.4%)	16 (29.6%)	
	Academic (grades 13–16)	17 (5.3%)	10 (3.8%)	7 (13%)	
Family member size	1–4 number	161 (50.3%)	141 (53%)	20 (37%)	0.037
	More than 4 number	159 (49.7%)	125 (47%)	34 (63%)	
Health insurance	No	69 (21.6%)	62 (23.3%)	7 (13%)	0.104
	Yes	251 (78.4%)	204 (76.7%)	47 (87%)	
Family history of PCa	No	298 (93.1%)	252 (94.7%)	46 (85.2%)	0.018
	Yes	22 (6.9%)	14 (5.3%)	8 (14.8%)	

Logistic regression (backward stepwise method) was performed to explain the demographic variable related to PSA test uptake (yes, no), and the best model was selected in the 2th step. Among the demographic variable, age, education level, economic status, family member size, health insurance and positive history of PCa were the most influential predictive factors related to PSA test uptake (Table 3).

**Table 3:** Multiple logistic regression results for demographic variable related to PSA test uptake

		B	S.E.	Wald	P	OR	95% C.I	
							Lower	Upper
Step 1	Age	0.126	0.042	8.861	0.003	1.134	1.044	1.232
	Marital status	1.116	0.696	2.572	0.109	3.053	0.780	11.941
	Education	0.849	0.194	19.188	< 0.001	2.338	1.599	3.420
	Economic	0.675	0.287	5.521	0.019	1.963	1.118	3.447
	Family member size	0.909	0.354	6.583	0.010	2.481	1.239	4.967
	Health insurance	1.020	0.488	4.368	0.037	2.772	1.065	7.213
	Family history of PCa	1.886	0.570	10.939	0.001	6.595	2.157	20.171
	Constant	−17.727	3.763	22.198	< 0.001	< 0.001		
Step 2	Age	0.103	0.040	6.695	0.010	1.108	1.025	1.198
	Education	0.882	0.193	20.809	< 0.001	2.415	1.653	3.527
	Economic	0.683	0.286	5.704	0.017	1.980	1.130	3.470
	Family member size	0.906	0.352	6.633	0.010	2.475	1.242	4.932
	Health insurance	0.991	0.482	4.232	0.040	2.693	1.048	6.920
	Family history of PCa	1.900	0.564	11.339	0.001	6.687	2.213	20.209
	Constant	−14.131	3.054	21.405	< 0.001	< 0.001		

Table 4 shows the Zero-order correlations. Significance levels at the 0.01 and 0.05 were the criteria for the analysis. The bivariate assessment of variables revealed that there were signs of multicollinearity among HBM variables.

**Table 4:** Bivariate correlation between predictor determinants of HBM

Determinants	Mean (SD)	Range	X1	X2	X3	X4	X5
X1. Perceived susceptibility	10.39 (2.22)	3–15	1				
X2. Perceived severity	17.39 (3.36)	5–25	0.515^**^	1			
X3. Perceived benefits	13.83 (3.46)	4–20	0.435^**^	0.254^**^	1		
X4. Perceived barriers	9.95 (3.37)	4–20	−0.417^**^	−0.329^**^	−0.119^*^	1	
X5. Perceived self-efficacy	9.56 (2.23)	3–15	0.340^**^	0.244^**^	0.186^**^	−0.443^**^	1
X6. Cues to action	1.16 (1.36)	0–4	0.384^**^	0.281^**^	0.209^**^	−0.325^**^	0.254^**^

** *P* <  0.01 *P <  0.05

Logistic regression analysis and backward stepwise method was used for calculating the predictability of HBM determinants on PSA test uptake (Table 5). As mentioned in statistical analyses, a step-wise model building procedure was conducted and finally on step 4 the procedure stopped and the best model was selected. The best determinants predictors for PSA test uptake were cues to action [OR: 1.967 and 95% CI: 1.546, 2.504], perceived severity [OR: 1.140 and 95% CI: 1.008, 1.290], and perceived benefits towards PSA test uptake [OR: 1.133 and 95% CI: 1.024, 1.253].

**Table 5:** Multiple logistic regression analysis for determinants of HBM related to PSA test uptake

	B	S.E.	Wald	P	OR	95% C.I	
						Lower	Upper
*Step 1*							
Benefits	0.187	0.063	8.818	0.003	1.205	1.066	1.364
Barriers	−0.073	0.070	1.091	0.296	0.930	0.810	1.066
Susceptibility	−0.261	0.140	3.500	0.061	0.770	0.585	1.013
Severity	0.223	0.091	6.044	0.014	1.249	1.046	1.492
Self-efficacy	0.008	0.078	0.010	0.921	1.008	0.865	1.175
Cues to action	0.707	0.134	27.876	< 0.001	2.029	1.560	2.638
*Step 2*							
Benefits	0.187	0.063	8.936	0.003	1.206	1.067	1.364
Barriers	−0.075	0.067	1.277	0.258	0.928	0.814	1.057
Susceptibility	−0.261	0.139	3.495	0.062	0.771	0.586	1.013
Severity	0.223	0.090	6.069	.014	1.250	1.047	1.492
Cues to action	0.708	0.134	28.128	< 0.001	2.031	1.563	2.639
*Step 3*							
Benefits	0.175	0.061	8.230	0.004	1.191	1.057	1.343
Susceptibility	−0.217	0.133	2.665	0.103	0.805	0.620	1.045
Severity	0.229	0.090	6.448	0.011	1.258	1.054	1.502
Cues to action	0.745	0.131	32.284	< 0.001	2.106	1.629	2.723
*Step 4*							
Benefits	0.125	0.052	5.864	0.015	1.133	1.024	1.253
Severity	0.131	0.063	4.325	0.038	1.140	1.008	1.290
Cues to action	0.677	0.123	30.270	< 0.001	1.967	1.546	2.504

## Discussion

The aim of this study was to determine prevalence and determinants related to uptake PSA test among sample of Iranian elderly men based on the HBM. According to the result 16.9% of the participants had PSA test uptake at least once. Bello et al. in their study among urban community in North-Central Nigeria reported that only 7.1% of Nigerian men had taken the PSA screening test at least once [19]. So et al. [20] stated that 10% of Chinese men aged 50 or more had taken PSA test. Burns et al. [21] carried out a research on men aged 40 years and over old in Republic of Ireland and reported that 24% of the participants had uptake of PCa screening. Furthermore, Ojewola et al. [22] in their study among 305 community-dwelling men older than 40 years in Southwest Nigeria indicated only 10.2% of them had taken the PSA screening test at least once. Furthermore, Carrasco-Garrido et al. carried out a research in Spain people and reported that the uptake PSA was 35.19% [23]. A review of these studies indicated that PSA test uptake is lower among Asian men compared to European men. In this regards, Consedine et al. stated that the likely variations in screening behavior among ethnic populations [24]. These findings can be warning to health policy makers in Asian country; and should be the focus of special attention.

The results of our study suggest that the following five demographic factors were related to the PSA test uptake among the Iranian elderly men: 1) increase age, 2) better economic status, 3) higher education level, 4) positive family history of prostate cancer, and 5) increase family member size. These results are generally consistent with the findings reported by other studies. For example, Merrill [25] in their study on 1293 men age 40 years or older in Utah reported that PSA screening significantly increased with age: 23.9% for ages 40–49, 51.4% for ages 50–59, 67.4% for ages 60–69, and 67.0% for ages 70+. Mirzaei-Alavijeh et al. [3] also conducted a study among men in western Iran and reported similar findings towards positive correlation between increased age and PCa screening tests. It seems that younger people perform less screening behaviors as they less often see themselves at risk.

In line with our finding the impact of the economic status on cancer screening behaviors has been shown in numerous studies [26–29]. In this regards, Guessous et al. [26] carried out research on 12,034 Swedish men aged ≥50 years (mean age: 63.9) and indicated men belonging to high socioeconomic status are significantly more frequently PCa screened than those less favored. Thus, higher economic level could lead to higher medical care such as screening test uptake. A national health insurance scheme may be necessary to increase PCa screening test uptake among Iranian men.

Our findings also indicated that the PSA test uptake is combined with the higher education level, which is in line with the findings of earlier studies towards investigating the factors related with cancer screening test [26, 30]. Kangmennaang et al. [30] in their study on 1244 men aged 40 and above in Namibia showed that higher education level (OR = 2.02) were more likely to screening for PCa.

Another finding of the current study was more PSA test uptake among men with a family history of PCa compared to men without a family history of PCa. This high level of PSA test uptake among men with a family history of PCa compared to men without a family history of PCa is consistent with observations from other studies. For example, Shah et al. [31] in National Health Interview Survey among male in United States reported that compared to men without a family history of PCa, men with a family history were more likely to uptake PSA. As well as, national guidelines for cancer screening emphasize screening tests for people with a family history of cancer [32]. Furthermore, having a family history of cancer may increase one understands of cancer and susceptibility of getting cancer motivate one to participate in screening [33]. The impact of family history on cancer screening tests may be attributed to health care providers’ recommendations for screening tests, increased knowledge of participants due to family history of cancer, or perhaps both.

The men who had family member size more than four had PSA test uptake more than other men. This result is similar to the results reported by other studies [3, 34]. Social support for receiving PCa screening test is often provided by one’s social network of family and friends [34]. It seems that involving families in health programs could have beneficial results for improving the society health status.

The results of the our study indicate that the perceived benefits towards the PSA test uptake, the perceived severity of PCa, and the cues to action towards the PSA test uptake, as the three main determinants of HBM, were associated with the Iranian elderly men to PSA test uptake. In the field of cancer screening test uptake, many studies have underlined the predictive potential of benefits, severity, and cues to action for uptake screening test by men ([20, 35, 36], and). Consequently, the results confirm suggestions that the HBM is a suitable theoretical basis for develop of the cancer screening promotion programs [12–17].

The perceived severity is a main fear arousal factor in explaining the behavior while people believe that they are vulnerable to get a disease [37]. Our results indicated that perceived severity of was important factor that mediate behavior to uptake PSA. Bloom et al. [35] carried out a research on 208 African American men, aged 40 to 74 years in California and indicated the positive significant associated with perceived risk and uptake PSA. Rundle et al. reported that changes in perceived PCa risk was mediator for promoting effectiveness of the PCa screening test promotion programs [28]. It seems that development of educational programs to increase seriousness about side effect of PCa could be beneficial of the results in order to PCa screening test promotion programs.

Perceived benefit refers to an individual’s assessment of the positive outcomes that are caused by a specific action [38]. Our findings showed that men who had higher perceived benefits towards PSA test uptake (OR = 1.13) was more likely to PSA test uptake. In line with our study, Avery et al. [36] in their study indicated perceived beliefs towards benefits of cancer screening can predict PSA test uptake.

According to our results, cues to action was strongest determinant was predictor PSA test uptake among the Iranian elderly men. The results of similar to studies confirm these finding [20, 39] and highlight the effectiveness of the health care workers in persuasion the men to uptake PCa screening program. For example, So et al. [20] carried out a study on 1002 men over than 50 years old in Hong Kong and reported health professionals recommendations was the strongest relationship with the PSA test uptake. Thus, health care workers can important role in the increase of cancer screening behaviors in Iranian elderly men. It seems that health care workers explaining the potential benefits of PSA testing can play an important role in promoting this test among Iranian men.

The findings reported in this study have certain limitations. First, data collection based on self-reporting, which always faces the risk of recall bias and we do not know how it could have affected the results. Second, high rejection rate is another limitation of our study. Finally, data collection only among sample of Iranian elderly men in the west of Iran and results cannot be generalized to other population of elderly men.

## Conclusion

There are multiple determinates to explain the cancer screening test uptake among elderly people. The current study confirmed the applicability of the HBM to explain PSA test uptake among elderly men in Iran. We conclude that we found there is some support to use the HBM to develop health promotion programs to improve PSA screening test uptake. In the other words, our result could be beneficial for guiding practitioners and health educators to develop evidence based promotion programs to increase PSA test uptake. Thus, HBM-based assessments of behavior may provide insights for intervention to modify and improve individuals’ beliefs towards benefits of PSA test uptake. Moreover, it seems that development of health promotion programs to increase cues to action and positive beliefs toward PSA test uptake and also perceived treat about side effect of PCa could be beneficial to increase PSA test uptake. Also, health care workers advice had an important role in persuading to PSA test uptake.

## Supplementary information

**Additional file 1:** **Supplementary file 1.** Questionnaire

## Data Availability

Please contact the corresponding author for data requests.

## Abbreviations

HBM: Health Belief Model
PCa: Prostate cancer
PSA: Prostate Specific Antigen
OR: Odds Ratio
SD: Standard Deviation
SPSS: Statistical Package for Social Sciences
