# Supplementary material for: Prostate specific antigen test uptake: a cross sectional study on elderly men in Western Iran
Source: BMC Geriatr. 2020 Aug 24;20:298. doi: 10.1186/s12877-020-01710-9 (PMC7444239; doi:10.1186/s12877-020-01710-9)
Supplement: Supplementary file 1 — Additional file 1: Supplementary file 1. Questionnaire [file 12877_2020_1710_MOESM1_ESM.docx]

**Supplementary file 1: Questionnaire**

**Dear citizen;**

**Greetings and Regards**

This questionnaire is designed to conduct a research project on the beliefs related to PSA screening uptake among the elderly men; your participation in this research is completely voluntary. All information collected from you in this study will be kept strictly confidential. Please help us in this study by providing your honest answers if you wish.

**Thank you in advance for your sincere cooperation.**

**Section I (Background Characteristics)**

**Instructions: Please complete the following items to the best of your ability.**

1. **Age:** ………….. years

2. **Marital Status**: Single □ Married □

3. **Economic Status**: Weak□ Middle□ Good□

4. **Level of Education**: Primary School □ Secondary School □ High School□ Academic □

5. **Family Member Size**: 1-4 number □ More than 4 number□

6. **Health Insurance**: Yes □ No □

7. **History of a Family Person who has had Prostate Cancer (PCa)**:Yes□ No□

**Section 2 (HBM Theoretical Determinants)**

**The following questions ask you to respond on a 5 point scale. Please place an X on where you feel best describes your response to the statement listed below about attending a PSA test uptake:**

| **No** | **Item** | **Strongly Disagree** | **Disagree** | **Undecided** | **Agree** | **Strongly Agree** |
| --- | --- | --- | --- | --- | --- | --- |
|  | **Perceived benefits towards the PSA test uptake** |  |  |  |  |  |
| 1 | PSA test uptake will help to diagnose PCa early. |  |  |  |  |  |
| 2 | PSA test uptake will help me not worry as much about PCa. |  |  |  |  |  |
| 3 | PSA test uptake will decrease my chances of dying from PCa. |  |  |  |  |  |
| 4 | PSA test uptake will help me to have a plan for the future about PCa. |  |  |  |  |  |
|  | **Perceived barriers related to PSA test uptake** |  |  |  |  |  |
| **1** | PSA test uptake is time-consuming. |  |  |  |  |  |
| **2** | I'm afraid of diagnose PCa. |  |  |  |  |  |
| **3** | Health center is far from my house to receive PSA test uptake. |  |  |  |  |  |
| **4** | PSA test uptake is too embarrassing. |  |  |  |  |  |
|  | **Perceived susceptibility** |  |  |  |  |  |
| **1** | It is likely that I will get PCa in the future. |  |  |  |  |  |
| **2** | My chances of getting PCa in the next few years are high. |  |  |  |  |  |
| **3** | I feel I will get PCa sometime during my life. |  |  |  |  |  |
|  | **Perceived severity** |  |  |  |  |  |
| **1** | PCa could seriously affect in my social life. |  |  |  |  |  |
| **2** | PCa imposes huge economic costs on my family. |  |  |  |  |  |
| **3** | PCa can kill me. |  |  |  |  |  |
| **4** | PCa is a serious disease. |  |  |  |  |  |
| **5** | Death from PCa is rare. |  |  |  |  |  |
|  | **Perceived self-efficacy** |  |  |  |  |  |
|  | *How confident are you that you can …* |  |  |  |  |  |
| **1** | Make an appointment to have a PSA test uptake? |  |  |  |  |  |
| **2** | Find the time to have a PSA test uptake? |  |  |  |  |  |
| **3** | Get a PSA test uptake even if you are worried about the results? |  |  |  |  |  |
|  | **Cues to action** |  |  |  |  |  |
| **1** | Doctors advised me to uptake PSA. |  |  |  |  |  |
| **2** | Health care workers encourage me to PSA test uptake. |  |  |  |  |  |
| **3** | My family encourages me to PSA test uptake. |  |  |  |  |  |
| **4** | How much the PCa death in others affects you to PSA test uptake? |  |  |  |  |  |

**Section 3 (PSA test uptake questionnaire)**

1. **Have you PSA test uptake at during last year**: Yes □ No □

***Good luck***
